# Chromosome instability and carcinogenesis: Insights from murine models of human pancreatic cancer associated with BRCA2 inactivation

**DOI:** 10.1016/j.molonc.2013.10.005

**Published:** 2013-11-06

**Authors:** Liam D. Cassidy, Siong-Seng Liau, Ashok R. Venkitaraman

**Affiliations:** ^1^ University of Cambridge, Medical Research Council Cancer Cell Unit, Hutchison/MRC Research Centre, Hills Road, Cambridge CB2 0XZ, United Kingdom

**Keywords:** Pancreatic cancer, BRCA2, Genetically engineered mouse model, Chromosomal instability, Hereditary cancer predisposition

## Abstract

Chromosomal instability is a hallmark of human cancer cells, but its role in carcinogenesis remains poorly resolved. Insights into this role have emerged from studies on the tumour suppressor BRCA2, whose inactivation in human cancers causes chromosomal instability through the loss of essential functions of the BRCA2 protein in the normal mechanisms responsible for the replication, repair and segregation of DNA during cell division. Humans who carry heterozygous germline mutations in the BRCA2 gene are highly predisposed to cancers of the breast, ovary, pancreas, prostate and other tissues. Here, we review recent studies that describe genetically engineered mouse models (GEMMs) for pancreatic cancer associated with BRCA2 mutations. These studies not only surprisingly show that BRCA2 does not follow the classical Knudson “two hit” paradigm for tumour suppression, but also highlight features of the interplay between TP53 inactivation and carcinogenesis in the context of BRCA2 deficiency. Thus, the models reveal novel aspects of cancer evolution in carriers of germline BRCA2 mutations, provide new insights into the tumour suppressive role of BRCA2, and establish valuable new preclinical settings for testing approaches to pancreatic cancer therapy; together, these features emphasize the value of GEMMs in cancer research.

## Highlights

- The mechanisms underlying pancreatic carcinogenesis in germline BRCA2 mutation carriers remain unclear.
- Recent studies on transgenic mouse models surprisingly show that even heterozygous BRCA2 mutations promote pancreatic cancer.
- They also reveal the interplay between TP53 inactivation and pancreatic carcinogenesis in the context of BRCA2 deficiency.
- We review insights into tumour evolution arising from these models, and highlight their value in testing new therapies.

Aberrations in the number and structure of chromosomes are a hallmark of cells derived from solid tumours. These aberrations not only include structural anomalies such as gross chromosomal rearrangements (including translocations, large deletions or inversions) and gene copy number variations, which have been connected to defective DNA replication or repair, but also anomalous chromosome number triggered by defective segregation between daughter cells during mitosis. Whereas much recent work has extensively documented the mechanisms underlying normal DNA replication, repair and segregation, what happens when these mechanisms are perturbed by the genetic alterations associated with cancer remains far less studied. Here, we will review this question from the perspective of recent work using transgenic models designed to recapitulate a specific human cancer involving mutations affecting the tumour suppressor gene *BRCA2*, associated with hereditary predisposition to breast, ovarian, pancreatic and other cancers. There is by now clear evidence to implicate the large, 3418 residue BRCA2 protein (3328 residues in the mouse) in the maintenance of chromosome integrity during cell division (Venkitaraman, 2009). BRCA2‐deficient cells accumulate structural chromosome aberrations as they divide, and also become aneuploid through losses or gains in whole chromosomes (Gretarsdottir et al., 1998; Patel et al., 1998; Tutt et al., 1999; Yu et al., 2000). A large body of evidence connects the chromosomal instability observed in BRCA2‐deficient cells to essential biological functions of the BRCA2 protein in DNA repair by homologous recombination (Connor et al., 1997; Patel et al., 1998; Moynahan et al., 2001), in progression through the S and G2/M phases of the cell cycle (Lomonosov et al., 2003; Ayoub et al., 2009; Menzel et al., 2011; Schlacher et al., 2011) and in mitotic cell division by cytokinesis (Daniels et al., 2004; Mondal et al., 2012). Thus, murine models that recapitulate the effect of cancer‐associated mutations in human *BRCA2* on tissue‐specific carcinogenesis provide an important opportunity to dissect the complex roles played by chromosomal instability during human carcinogenesis.

## 1 Modelling human pancreatic cancer associated with *BRCA2* inactivation

Pancreatic ductal adenocarcinoma (PDAC) represents the fourth leading cause of cancer mortality worldwide, with an incidence of approximately 217,000 new cases each year nearly matched by 213,000 deaths (Parkin et al., 2001). Several of the most frequent genetic events underlying the initiation and progression of human pancreatic cancer have been identified (Hezel et al., 2006; Maitra and Hruban, 2008). These include activating mutations in the *KRAS* proto‐oncogene, which occur in >90% of PDAC (Caldas and Kern, 1995) and are considered as a key driver for pancreatic carcinogenesis, and mutations inactivating the *TP53* gene, which occur in 50–75% of patients (Redston et al., 1994).

Moreover, several lines of evidence implicate mutations inactivating the *BRCA2* tumour suppressor in an estimated 5–20% of familial PDAC (Hahn et al., 2003; Couch et al., 2007). Germline carriers of deleterious *BRCA2* mutations that commonly truncate the encoded protein exhibit an increased lifetime risk of developing PDAC, in addition to their well‐known predisposition to cancers of the breast and ovary (Breast Cancer Linkage Consortium, 1999). Within high‐risk pancreatic cancer kindreds, inherited mutations in *BRCA2* represent the most frequently encountered germline genetic alteration (Hahn et al., 2003). The incidence of germline *BRCA2* mutations in apparently sporadic pancreatic cancers may be as high as in breast or ovarian cancer (Goggins et al., 1996). More recently, *PALB2*, which encodes a BRCA2‐interacting protein also essential for homology‐directed DNA repair, has emerged as a pancreatic cancer susceptibility allele (Jones et al., 2009).

**Figure 1:**
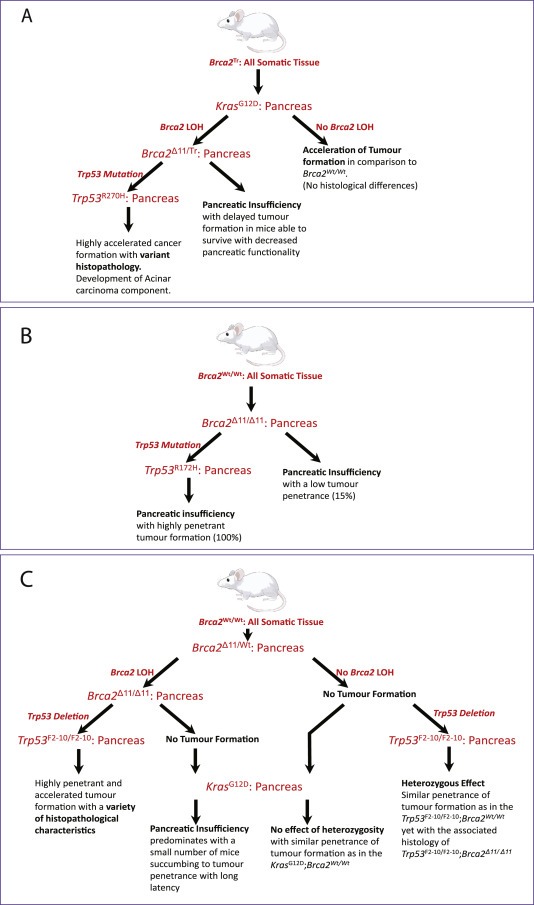
Modelling the role of Brca2 in PDAC development. Key features and findings from the three genetically engineered mouse models (GEMMs) are summarized here, and described in the main text. (A) Skoulidis et al. uniquely employ a truncated Brca2Tr allele mimicking germline mutations in human mutation carriers expressed in all somatic tissues. In contrast, (B) Feldmann et al. and (C) Rowley et al. conditionally delete Brca2 in the pancreas alone. All three GEMMs introduce tissue‐specific alleles activating Kras or inactivating Trp53. It is important to note that the Brca2 alleles as well as the Trp53 alleles used in each model are distinct.

Three new transgenic models for pancreatic adenocarcinoma associated with *BRCA2* inactivation have recently been described (Skoulidis et al., 2010; Feldmann et al., 2011; Rowley et al., 2011) [Figure 1]. One of these models does not incorporate activation of the *Kras* oncogene (Feldmann et al., 2011). In contrast, the other two models (Skoulidis et al., 2010; Rowley et al., 2011) use a conditional gene‐targeted allele developed by Tuveson, Jacks and colleagues (Jackson et al., 2001; Johnson et al., 2001), in which tissue‐specific activation of oncogenic *Kras*G12D is driven on a single allele by *loxP*‐CRE mediated recombination, mimicking a genetic event that frequently triggers *Kras* activation in human cancers. CRE recombinase expression is controlled by the PDX1 promoter, which is expressed at E8.5 and required for organogenesis of the pancreas, whereby loss of the gene is associated with an absence of pancreatic formation (Jonsson et al., 1994; Offield et al., 1996). The expression of the PDX1‐CRE transgene therefore occurs throughout the pancreatic cellular compartment, albeit in a stochastic manner, to trigger *Kras* activation (Hingorani et al., 2003).

Patients who carry germline mutations affecting *BRCA2* harbour the germline mutant allele in all somatic tissues, whereas the second *BRCA2* allele is wildtype (Wooster et al., 1994). It has been widely believed that loss of the second, wild‐type *BRCA2* allele in nascent cancer cells (termed ‘loss of heterozygosity’ or LOH) is necessary for the emergence of tumours in germline mutation carriers. The pancreatic cancer model developed in our laboratory (Skoulidis et al., 2010) mimics this presumed sequence of events. It carries in all somatic tissues a truncated allele of murine *Brca2* (*Brca2* ^*Tr*^), which truncates the gene in an evolutionarily conserved and functionally critical region encoded by exon 11, resembling deleterious germline mutations found in human carriers (Friedman et al., 1998). The second *Brca2* allele, *Brca2* ^*F11*^ (Jonkers et al., 2001) can be conditionally disrupted to remove exon 11 by *loxP*‐CRE recombination in the pancreas. This event is driven by PDX1‐CRE, and therefore occurs in the same tissues which undergo *Kras* activation. There is evidence that both the *Brca2* ^*Tr*^ and *Brca2* ^*F11*^ alleles can express a truncated protein product (Patel et al., 1998; Choi et al., 2012), a point we will later return to. Notably, the models developed by Rowley et al. and Feldmann et al. both exclusively use a strain homozygous for the conditional *Brca2* ^*F11*^ allele; thus, germline heterozygosity for *BRCA2* is not modelled in their experiments.

All three of the new pancreatic cancer models incorporate conditional alleles that inactivate *Tp53* in the pancreas, to mimic the frequent loss of this tumour suppressor in human pancreatic cancers. Somewhat different *Tp53* alleles are used by each group, an important distinction given that the nature of *Tp53* mutations is thought to affect pancreatic cancer development (Olive et al., 2004). Two of the studies employ gain‐of‐function mutants affecting a single allele. These are either the structural mutant Tp53^R172H^ (Feldmann et al., 2011) or the contact mutant Tp53^R270H^ (Skoulidis et al., 2010). Both these *Tp53* mutants are associated with the development of carcinomas (Olive et al., 2004). In contrast, the third study uses a null allele of *Tp53*, wherein exons 2–10 are deleted by PDX1‐CRE activation (Rowley et al., 2011), in a manner that less faithfully represents human cancer‐associated mutations.

Thus, it should be clear from the foregoing that these three recently‐published models for pancreatic carcinogenesis associated with *BRCA2* inactivation harbour important differences not only in the tissue‐specificity, nature and timing of mutant *Tp53* and *Brca2* alleles, but also in the presence of mutant *Kras*. We believe that these distinctions are vital to understanding the marked differences in pancreatic carcinogenesis observed in the studies, the key findings from which are highlighted in Table 1.

**Table 1:** Comparison of the major phenotypes associated with Brca2‐deficient PDAC in three GEMMs. Each model employs distinct Brca2 alleles, in the context of different initiating lesions. Possible mechanisms underlying the variations in phenotype are discussed in the main text.

Study	Cohort	Associated phenotype
Skoulidis et al., 2010	*Kras^G12D^*	15% tumour penetrance with long latency; 100% PDAC
	*Kras^G12D^, Brca2^Tr/Wt^*	Accelerated tumourigenesis with an increase in tumour penetrance at 30%; 100% PDAC
	*Kras^G12D^, Brca2^Tr/F11^*	Pancreatic insufficiency; some tumours develop but with long latency; 100% PDAC
	*Kras^G12D^, Tp53^R270H^, Brca2^Wt/Wt^*	Highly penetrant tumour formation with a median survival of 168 days; 100% PDAC
	*Kras^G12D^, Tp53^R270H^, Brca2^Tr/Wt^*	Accelerated tumourigenesis, median survival 143 days, in comparison to Brca2Wt; 100% PDAC
	*Kras^G12D^, Tp53^R270H^, Brca2^Tr/F11^*	Further acceleration of tumourigenesis, median survival 84 days, all tumours showed regions of PDAC development with noted regions of Acinar histology in 18% of cases
Rowley et al., 2011	*Kras^G12D^*	61% tumour penetrance with median survival 406 days
	*Kras^G12D^, Brca2^F11/Wt^*	66% tumour penetrance with median survival 366 days‐ similar to *Brca2^Wt^*
	*Kras^G12D^, Brca2^F11/F11^*	Pancreatic insufficiency; 13% tumour penetrance with long latency
	*Brca2^Wt/Wt^*	No tumour formation
	*Brca2^F11/Wt^*	No tumour formation
	*Brca2^F11/F11^*	No tumour formation
	*Tp53^F2‐10/F2‐10^, Brca2^Wt/Wt^*	Low tumour penetrance with acinar histology
	*Tp53^F2‐10/F2‐10^, Brca2^F11/Wt^*	Similar penetrance of tumour formation to Brca2Wt but with the associated histology of *Brca2^F11/F11^*
	*Tp53^F2‐10/F2‐10^, Brca2^F11/F11^*	Increased tumour penetrance and acceleration of tumour formation in comparison to *Brca2^Wt^* of *Brca2^F11/Wt^* Mixed histology: PDAC 40%, Acinar 15%, high‐grade undifferentiated 35%, remainder mucinous tumours. Median survival 300 days.
Feldmann et al., 2011	*Brca2^F11/F11^*	Pancreatic insufficiency; development of PDAC but with incomplete penetrance (∼15%) at 15 months. Median survival 454 days.
	*Tp53^R172H^, Brca2^F11/F11^*	Pancreatic insufficiency; highly penetrant PDAC formation (100%) at 15 months. Median survival 375 days.

## 2 *Brca2*^*Tr*^ heterozygosity suffices for pancreatic carcinogenesis driven by mutant *Kras*

BRCA2 has been believed to follow the classical ‘two‐hit’ paradigm for tumour suppression (Smith et al., 1992; Collins et al., 1995; Rahman and Stratton, 1998). Initial studies soon after the discovery of *BRCA2* reported consistent inactivation of the wild‐type *BRCA2* allele through loss‐of‐heterozygosity (LOH) in breast or ovarian cancer cells from mutation carriers (Collins et al., 1995; Gudmundsson et al., 1995), engendering the widely accepted view that *BRCA2* LOH is an essential event in carcinogenesis. A few notes of dissent have emerged in later studies (King et al., 2007; Willems et al., 2008), but they have not gained widespread attention.

In this context, it is notable that the studies reported in Skoulidis et al. unexpectedly reveal that *BRCA2* heterozygosity promotes pancreatic cancer development in mice and men. In both the *Tp53* wildtype and *Tp53* ^*R270H*^ cohorts from the murine model, heterozygosity for Brca2 (through the *Brca2* ^*Tr/Wt*^ genotype) acts with *Kras* ^*G12D*^ to accelerate the progression and development of PDAC. A similar conclusion is reached from studies on a small number of human pancreatic cancer samples from carriers of the Icelandic founder mutation in *BRCA2*, the allele *BRCA2* ^*999Del5*^, which is 5 bp deletion in exon 9 that causes a frame‐shift leading to the expression of a very short and unstable protein product (Mikaelsdottir et al., 2004). Three of the 4 cases tested do not exhibit LOH.

How heterozygosity for *Brca2* ^*Tr*^ may promote tumourigenesis remains uncertain. One possibility is that this genotype causes a mutator phenotype, owing to defects in DNA repair arising from a known role of BRCA2 in homologous DNA recombination (Patel et al., 1998; Moynahan et al., 2001; Tutt et al., 2001). However, previous studies on murine embryo fibroblasts (MEFs) heterozygous for *Brca2* ^*Tr*^ reveal no statistically significant effects on sensitivity to genotoxic agents (Patel et al., 1998; Yu et al., 2000). Neither *Brca2* ^*Tr/WT*^ mice (Friedman et al., 1998), nor strains heterozygous for other *Brca2* truncation mutants (Connor et al., 1997; Sharan et al., 1997; Jonkers et al., 2001; Yan et al., 2004), exhibit cancer predisposition. Notably, a *lacZ* mutation reporter gene (Boerrigter et al., 1995) incorporated into the germline of mice heterozygous for a *Brca2* truncation similar but not identical to *Brca2* ^*Tr*^ (Tutt et al., 2002) reveals no evident mutator phenotype. On the other hand, MEFs from this strain showed a mild alteration in DNA repair kinetics during recovery from 4Gy of ionizing radiation. Thus, there is little convincing evidence that heterozygosity for these *Brca2* mutant alleles creates a DNA repair defect that could explain heightened cancer predisposition, although the possibility has not yet been conclusively excluded.

In this connection, it is important to note that these cellular approaches do not yet account for the cooperative effect of mutant *Kras* on pancreatic carcinogenesis associated with *Brca2* heterozygosity, as suggested by the murine model developed by Skoulidis et al. Even a subtle increase in mutational load induced by *Brca2* heterozygosity in mutant *Kras* expressing cells – which might be undetectable in cellular experiments, but significant *in vivo* – could plausibly accelerate the progression of pre‐malignant pancreatic intra‐epithelial (PanIN) lesions (which occur frequently even in apparently normal pancreatic parenchyma (Hruban et al., 2008)) to overt malignancy. Further studies addressing this issue in murine models are clearly warranted.

Whether different *BRCA2* alleles behave in a manner similar to *Brca2* ^*Tr*^ is not clear. Like *Brca2* ^*Tr*^, heterozygosity for *BRCA2* ^*999Del5*^ apparently suffices to predispose human carriers to pancreatic carcinogenesis. However, the instability of the truncated protein encoded by *BRCA2* ^*999Del5*^ (Mikaelsdottir et al., 2004) suggests that haploinsufficiency for *BRCA2* (as opposed to any *trans*‐dominant effect of a mutant BRCA2 protein) accounts for the phenotypic effects of heterozygosity in patients who carry this Icelandic founder mutation. In contrast, Rowley et al. describe no heterozygous effect in any of their *Brca2* ^*F11/Wt*^ cohorts despite the presence of mutant *Kras* ^*G12D*^. Interpretation of this difference is not straightforward, since the *Brca2* ^*F11*^ allele engenders *Brca2* loss only in the cells expressing PDX1‐CRE, unlike *Brca2* ^*Tr*^, which is expressed in all somatic cells. This raises the possibility that non‐cell autonomous effects of *Brca2* ^*F11*^ heterozygosity – for example on stromal cells rather than the nascent cancer cells – may account for the cancer‐predisposing effect of the *Brca2* ^*Tr*^ allele.

Mitotic functions have also been ascribed to BRCA2, and interestingly, defects in G2 checkpoint function (Menzel et al., 2011), mitotic checkpoint enforcement (Choi et al., 2012) and the completion of cell division by cytokinesis (Daniels et al., 2004; Shive et al., 2010; Jonsdottir et al., 2012; Mondal et al., 2012) have been reported in *BRCA2*‐deficient cells. Whether or not these roles for BRCA2 may explain the effect of heterozygosity in tumour development is yet to be explored. Heterozygosity for the *Brca2* ^*Tr*^ allele is enough to trigger cytokinetic defects in MEFs (Daniels et al., 2004), but it is unclear whether the other mitotic functions are perturbed by *BRCA2* heterozygosity.

Importantly, recent data from human studies further support that *BRCA2* heterozygosity is enough to promote carcinogenesis. In breast cancers, incomplete loss of the remaining wild‐type allele has been observed using techniques more sensitive than those applied in the original studies (King et al., 2007). Importantly large‐scale, unbiased genomic sequencing of high‐grade serous ovarian carcinomas highlighted the retention of the wild‐type allele in end stage disease from ∼25% of germline *Brca2* carriers (Atlas, 2011). Furthermore, a detailed study of prostate tumour progression in *BRCA2* germline mutation carriers uncovered no LOH in high‐grade prostatic intraepithelial neoplasias, considered precursor lesions to the development of prostate adenocarcinoma, and up to 55% of the malignant tumours analysed (Willems‐Jones et al., 2012). Collectively, these data suggest that cancers arising in germline *BRCA2* mutation carriers frequently fail to exhibit loss of the wildtype allele, and that failure to exhibit LOH occurs in *BRCA2*‐mutant cancers from several different tissues. Thus, *BRCA2* may not follow the classical Knudson “two hit” paradigm for tumour suppression.

Interestingly, these conclusions can be set against the emerging backdrop of ongoing studies on tissue samples from patients with familial forms of pancreatic cancer. A study of 58 pancreatic intra‐epithelial neoplasms and intraductal papillary mucinous neoplasms reveals that somatic losses in *BRCA2* copy number are infrequent (Hong et al., 2012). However, definitive evidence addressing the extent to which the lessons from GEMMs of *Brca2*‐deficient pancreatic cancers can be applied to human neoplasia awaits the results of more extensive genome sequencing studies on pancreatic cancer samples from patients harbouring germline *BRCA2* mutations.

## 3 Pancreatic cancer histopathology and *BRCA2* genotype

Murine pancreatic cancers emerging in *Brca2* ^*Tr/F11*^ strains in which both *Brca2* alleles are inactivated in PDX1‐CRE expressing cells exhibit a preponderance of acinar cell carcinoma histology. Correspondingly, 3 of the four human pancreatic cancers from *BRCA2* ^*999Del5*^ mutation carriers that exhibited LOH were also of the acinar type (Skoulidis et al., 2010), which normally accounts for only 1–2%% of human pancreatic cancers (Hruban, 2007). This raises the possibility that these genotypes promote the evolution of acinar cell carcinomas rather than PDAC. Rowley et al. (2011) also observe differences in the histopathological spectrum of pancreatic malignancies from mice in which *Brca2* as well as *Tp53* had been inactivated, when compared to *Tp53* deficiency alone. These observations raise the possibility that the nature of *Brca2* mutations, their timing, or their coincidence with alterations with *Tp53* may alter the histopathological evolution of pancreatic cancers in mice. However, these observations remain too limited to allow firm conclusions to be drawn, and we draw attention to them here simply to emphasize the need for further studies.

## 4 Checkpoint inactivation, *Tp53* mutations, and the evolution of cancers following *Brca2* inactivation

We and others have shown (Patel et al., 1998; Lee et al., 1999; Tutt et al., 1999) that the genome‐wide DNA damage that follows homozygous inactivation of *BRCA2* leads to checkpoint activation and cell cycle arrest, rather than the unrestrained cellular proliferation typical of cancer. We have previously proposed (Venkitaraman, 2009) that checkpoint inactivation may therefore be an essential pre‐requisite for homozygous *BRCA2* inactivation through LOH during carcinogenesis. The work of Skoulidis et al. provides strong *in vivo* evidence for this hypothesis, supported by the observations of Rowley et al. In both murine models, bi‐allelic *Brca2* inactivation by itself leads to a loss of exocrine pancreatic parenchyma, a concomitant increase in adipose tissue, and progressive loss of organ functionality. Skoulidis et al. further demonstrate that pancreatic insufficiency is preceded by the widespread occurrence of DNA double‐strand breakage marked by γH2AX staining in cells lacking both copies of *Brca2*. Moreover, both Skoulidis et al. and Rowley et al. find that the concomitant inactivation of *Tp53* function prevents pancreatic insufficiency, and allows rapid PDAC development, in the pancreas of mice carrying bi‐allelic mutations inactivating *Brca2*. When the observations from these studies are synthesized, a picture emerges wherein *BRCA2* heterozygosity in germline mutation carriers may suffice to allow the development of *Kras*‐driven PDAC. Later inactivation of *Tp53* or other checkpoint genes may then allow eventual loss of the second *BRCA2* allele: although LOH is not an obligate step, it may promote the emergence of advanced cancers. Indeed, inferences from a very small study of just 5 samples from human pancreatic cancer patients support such a scenario, although it remains to be firmly established.

## 5 Mouse models for PDAC associated with *BRCA2* inactivation: lessons for cancer therapy

The work of Skoulidis et al. has implications for cancer therapy. As discussed above, our results suggest that *Brca2* heterozygosity suffices for PDAC formation driven by mutant *Kras* in mice and men. However, the rationale for the use of targeted agents such as PARP1 inhibitors (PARP1i) in *BRCA2*‐deficient cancers is contingent upon bi‐allelic *BRCA2* inactivation in the tumour cells (Bryant et al., 2005; Farmer et al., 2005). Therefore, as confirmed in our work (Skoulidis et al., 2010), PDAC cells that retain a functional *Brca2* allele are resistant to PARPi such as the AstraZeneca compound Olaparib. Thus, PARP1 inhibitors should be reserved for clinical use when *BRCA2* LOH can be verified in the tumour, assessment of which emerges as a critical requirement in the design of human clinical trials for the treatment of *BRCA2*‐deficient cancers.

These findings exemplify how the new generation of GEMMs for PDAC may represent valuable surrogate models for preclinical tests of therapeutic efficacy in patients. Importantly, such models not only allow *in vivo* proof of new therapeutic concepts, but may also provide a platform to assess the pharmacodynamic and pharmacokinetic properties of new agents, although species‐specific differences may limit such interpretations. The models also provide a flexible method to assess the impact of therapy on tumour progression using adapted multimodal imaging and drug bioavailability (including tissue drug penetrance) analyses. An important feature that determines if a particular GEMM is useful as a preclinical platform is if the model recapitulates a similar clinical response to standard therapy agents in clinical use in human. For instance, the KPC mouse model is relatively unaffected by gemcitabine similar to the small clinical benefit from this agent in the advanced pancreatic cancer setting in humans (Olive et al., 2009).

Each GEMM can be likened to a patient with a particular tumour type, and hence, can be enrolled into preclinical trial of novel agents (Eklund et al., 2013; Guerra and Barbacid, 2013). Such trials are facilitated by the use of adapted imaging techniques to monitor for tumour development and progression. Such utility is beginning to have an impact in the clinical setting. In humans, early phase clinical trials have shown promise for the combination of nanoparticle albumin‐linked paclitaxel (nab‐paclitaxel) and gemcitabine in advanced PDAC. Frese and colleagues have used the KPC mouse model of pancreatic cancer to provide a mechanistic understanding of the synergistic effect of this combination. Paclitaxel appears to inhibit the breakdown of gemcitabine through modulation of a degradative enzyme, cytidine deaminase through a reactive oxygen species‐dependent pathway (Frese et al., 2012). Such mechanistic analyses may help to rationalize our clinical strategy of using such drug combination. For instance, the nab‐paclitaxel can be used as an inducting agent followed by gemcitabine to enhance the tumouricidal effect of the latter.

Rational clinical trials in man are likely to benefit from the incorporation of an *in vivo* component that provides relatively rapid feedback of the predicted response to new agents. The preclinical assessment using GEMMs can either be used to screen potentially useful therapeutic agents before progressing to clinical trials, or alternatively, to critically assess the mechanisms of action *in vivo* once an agent has been found to be effective in a small‐scale trial, before progressing to larger Phase III clinical trial. One potential advantage of such GEMMs is that unlike human trials, they will allow sequential sampling of appropriate tumour tissues to assess the pharmacodynamic impact of a particular agent. In pancreatic cancer, several novel agents targeting a diverse range of molecular pathways have been tested in GEMMs to complement early phase clinical trials (Olive et al., 2009; Plentz et al., 2009; Cook et al., 2012; Jacobetz et al., 2012). The results of these trials will in due course affirm or refute the value of PDAC GEMMs as a predictive tool for clinical efficacy in human cancers.

The potential value of PDAC GEMMs as surrogates for the preclinical testing of new therapies is critically dependent on how closely these models mimic human PDAC. Several studies (Hingorani et al., 2003; Tuveson and Hingorani, 2005; Olive et al., 2009; Plentz et al., 2009; Cook et al., 2012) have emphasized the similarities in histopathology, cancer progression, clinical behaviour and even drug pharmacodynamics between PDAC GEMMs and human PDACs. However, it remains unclear whether the spectrum of genetic alterations is similar. Initial observations suggest that murine KPC PDACs bear resemblance to the human disease insofar as they exhibit a high degree of genomic instability, evident from multiple non‐reciprocal chromosomal translocations (Hingorani et al., 2005). However, with emerging data from large‐scale sequencing of human PDAC tumours (Biankin et al., 2012), it is now imperative that we further validate the GEMMs at the genomic level to compare the genomic landscapes of murine and human tumours.

Because PDAC GEMMs incorporate high‐penetrance genetic events such as initiating oncogenes or inactivated tumour suppressor genes from an early stage in a large number of susceptible cells, the resulting stereotypy of the malignancies arising therein may not reflect the heterogeneity likely to be present in human cancers. Importantly, the genetic heterogeneity of human cancers may give rise to differing therapeutic responses to any particular agent due to the differing genetic and epigenetic signatures of the constituent cells. It is conceivable that individual tumours can take differing genetic ‘routes’ to achieve tumoural progression, depending on the type of initiating genetic lesions and secondary genetic hits that occur stochastically. It is presumed that through inactivation of genes involved in maintaining genomic stability (e.g. *BRCA2* in models of pancreatic cancer) may promote the stochastic acquisition of genetic and consequent morphologic heterogeneity due to the expected increase in mutation rate. However, this point remains to be established in future studies, and also has important implications for the potential value of GEMM models in testing new therapeutic approaches against PDAC.
